# Life Review Depression Intervention Conducted by Asian and White American Caregivers: A Mixed‐Method Comparative Study

**DOI:** 10.1002/alz.089634

**Published:** 2025-01-09

**Authors:** Christina E. Miyawaki, Erin D. Bouldin, Angela McClellan

**Affiliations:** ^1^ University of Houston Graduate College of Social Work, Houston, TX USA; ^2^ University of Utah School of Medicine, Salt Lake City, UT USA

## Abstract

**Background:**

Approximately 10% of people living with Alzheimer’s dementia (PWD) experience depression, yet behavioral interventions remain scarce. We developed an innovative depression intervention, *Caregiver‐Provided Life Review (C‐PLR)* based on life review therapy. The purposes of this study were to evaluate the feasibility of training family caregivers in life review skills and evaluate the impact on depressive symptoms.

**Methods:**

Using a mixed‐method design, we trained Asian (n = 15) and White (n = 24) caregiver‐care recipient dyads. Eligible caregivers were family members who cared for PWD for ≥8 hours/week, completed caregiver training, six weekly life review sessions, and five weekly Fidelity check‐in calls with the research team. Care recipients experienced mild depression (≥5 on Geriatric Depression Scale) and dementia (≥13 on Telephone‐Montreal Cognitive Assessment). We collected pre‐ and post‐measures of care recipients’ depression (primary outcome), life satisfaction, caregivers’ burden, rewards of caregiving, and dyads’ relationship quality (secondary outcomes), and compared them using paired t‐tests. We evaluated feasibility by measuring fidelity check‐in scores to the C‐PLR training protocol during these calls.

**Results:**

Thirty‐nine dyads completed the study. Asian (mean age = 52 years old) and White (59 years old) caregivers were working, female, and in good‐excellent health. Their care recipients (Asian = 82 years old; White = 80) were female and in poor‐fair health. Both Asian and White care recipients’ depression and dyads’ relationship scores from baseline to the end of the study significantly improved (Asian = 4.1 to 2.1, *p*<0.001; 12.5 to 13.4, *p* = 0.029; White = 4.1 to 3.1, *p =* 0.049; 13.8 to 14.5, *p* = 0.044, respectively). Asian (73%) and White (75%) caregivers’ fidelity scores were similarly high (*p* = 0.91) and rated as good‐excellent. Qualitative interviews of both caregivers supported the sentiments of the quantitative results. Furthermore, 73% of Asian dyads conducted the C‐PLR in their native language, however, it did not appear to influence any outcomes.

**Conclusions:**

We found a significant impact of C‐PLR on depressive symptoms in both Asian and White PWD and their dyadic relationships. Both quantitative and qualitative results supported the feasibility of the C‐PLR training and the successful completion of the intervention. This study demonstrated that the C‐PLR intervention can be added to a list of successful non‐pharmaceutical interventions.

